# Multigland dysfunction from immune-checkpoint inhibitors: a case of hypothyroidism, diabetes, and adrenal insufficiency

**DOI:** 10.1210/jcemcr/luag024

**Published:** 2026-02-23

**Authors:** Ranjini Vengilote, Oyiyechukwu Onwudiwe, Alshaima Yousef, Michael Quartuccio

**Affiliations:** Department of Medicine, Rochester General Hospital, Rochester Regional Health, Rochester, NY 14621, USA; Division of Endocrinology, Diabetes, and Metabolism, Rochester Regional Health, Rochester, NY 14607, USA; Department of Medicine, Rochester General Hospital, Rochester Regional Health, Rochester, NY 14621, USA; Division of Endocrinology, Diabetes, and Metabolism, Rochester Regional Health, Rochester, NY 14607, USA

**Keywords:** immune-checkpoint inhibitors, nivolumab, ipilimumab, hypothyroidism, adrenal insufficiency, diabetes mellitus

## Abstract

Immune-checkpoint inhibitors (ICIs) such as nivolumab and ipilimumab have improved outcomes in metastatic renal cell carcinoma (RCC) but can cause immune-related adverse events (irAEs), including potentially irreversible endocrine toxicities with long-term treatment implications. We present a 62-year-old man with metastatic clear-cell RCC who developed 3 distinct endocrine irAEs during ICI therapy: thyroiditis evolving into hypothyroidism, insulin-dependent diabetes mellitus with diabetic ketoacidosis, and secondary adrenal insufficiency (AI), necessitating ICI discontinuation. Additionally, he experienced immune-mediated inflammatory arthritis. His course required thyroid hormone replacement, insulin therapy, and hydrocortisone for AI. This case underscores the potential for sequential, multiglandular endocrine toxicities from ICIs, a phenomenon infrequently reported in the literature. Clinicians should remain vigilant for delayed or evolving presentations, even in the absence of autoantibodies or radiographic abnormalities. Early recognition, multidisciplinary management, and long-term follow-up are critical for ICI-associated endocrinopathies.

## Introduction

Immune-checkpoint inhibitors (ICIs), such as the programmed cell death protein 1 (PD-1) inhibitor (nivolumab) and the cytotoxic T-lymphocyte-associated protein 4 (CTLA-4) inhibitor (Ipilimumab), have transformed the treatment landscape of metastatic renal cell carcinoma (RCC) by enhancing antitumor immune responses [1, 2]. However, these agents are associated with immune-related adverse events (irAEs), which may involve multiple organ systems. Endocrine irAEs, including thyroid dysfunction, diabetes mellitus, and adrenal insufficiency (AI), are particularly important because they are often irreversible and require lifelong hormone replacement [3-5].

Although individual endocrinopathies are well recognized, sequential development of multiple distinct endocrine irAEs in a single patient is rare, with only isolated reports of multiglandular involvement [6-9]. We describe a patient with metastatic RCC on ICI therapy who developed immune-mediated inflammatory arthritis along with a triad of endocrine complications: thyroiditis with subsequent hypothyroidism, insulin-dependent diabetes mellitus, and secondary AI.

## Case presentation

This is a case of a 62-year-old male with a medical history significant for clear-cell RCC of the left kidney, status post nephrectomy 10 years ago, with biopsy-proven metastasis to the body of the pancreas diagnosed 1 year before presentation. He was started on palliative ICI therapy with 4 cycles of nivolumab and ipilimumab every 21 days, followed by maintenance therapy with nivolumab alone every 2 weeks.

## Diagnostic assessment

Two months into ICI therapy, the patient presented to the oncology office for follow-up with an unexplained 10-pound weight loss. He was hemodynamically stable, and no thyroid swelling was noted on presentation. Outpatient laboratory studies revealed free thyroxine (FT4) of 6.3 ng/dL (81.1 pmol/L) (reference range: 0.9-1.8 ng/dL [SI: 11.6-23.2 pmol/L]), and thyroid-stimulating hormone (TSH) of 0.01 µIU/mL (0.01 mIU/L) (reference range: 0.55-4.78 µIU/mL [SI: 0.4-4.0 mIU/L]), consistent with subclinical hyperthyroidism. Subsequently, repeat outpatient laboratory studies after 1 month revealed FT4 of 0.2 ng/dL (SI: 2.57 pmol/L) and TSH of 33.38 µIU/mL, suggestive of hypothyroidism. Oral levothyroxine 100 mcg daily was initiated. Antithyroid peroxidase (anti-TPO) antibodies were normal at 44 U/mL (44 kU/L) (reference range: 0-60 U/mL) at that time.

Three months into the ICI therapy, he presented to the hospital with generalized arthralgia. Laboratory studies showed elevated acute-phase reactants, as evidenced by an erythrocyte sedimentation rate (ESR) of 87 mm/hour (reference range: 0-20 mm/hour) and a C-reactive protein (CRP) of 81.5 mg/L (reference range: 0-8.0 mg/L). Autoimmune workup, including rheumatoid factor (RF), antinuclear antibody (ANA), cyclic citrullinated peptide antibodies (anti-CCP), double-stranded deoxyribonucleic acid antibodies (Anti-dsDNA), and creatine kinase, was negative. The patient was diagnosed with immune-mediated inflammatory arthritis. He initially received intravenous dexamethasone 24 mg once daily and was subsequently discharged with a prolonged outpatient steroid taper using oral prednisone 50 mg twice daily. The steroid taper was discontinued after initiation of hydroxychloroquine and later methotrexate for symptom control.

**Figure 1:**
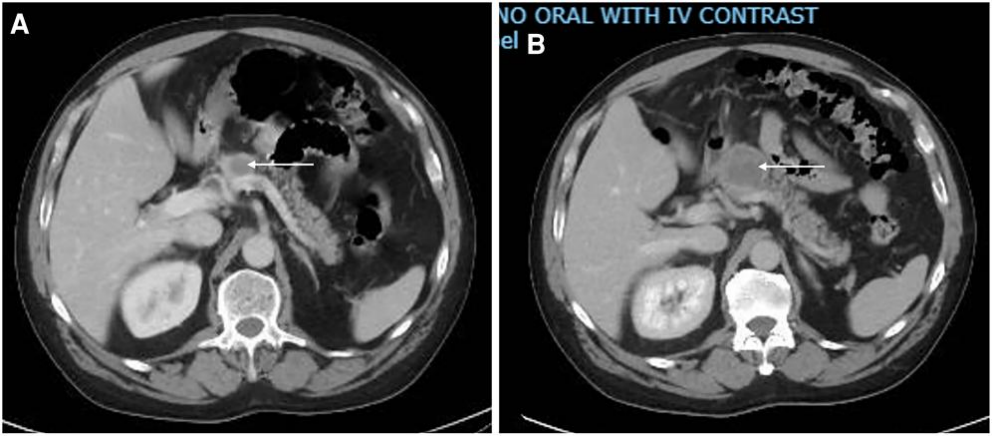
CT scan of the abdomen showing the 1.7 cm pancreatic mass (white arrow, panel A) decreased from 3.3 cm (white arrow, panel B).

Six months into the ICI therapy, he presented again with polyuria and polydipsia. Laboratory studies were notable for hyperglycemia of 458 mg/dL (SI: 25.4 mmol/L) (reference range: 60-140 mg/dL [SI: 4.0-5.6 mmol/L]), hemoglobin A1c (HbA1c) of 8.4% (SI: 68 mmol/mol) (reference range: 4.2-5.6% [SI: 20-42 mmol/mol]), and a C-peptide level of 2.4 ng/mL (0.79 nmol/L) (reference range: 0.8-3.8 ng/mL [SI: 0.26-0.62 nmol/L]). Antiglutamic acid antibodies were negative. Computed tomography (CT) scan of the abdomen showed a stable 1.7 × 1.5 cm pancreatic neck mass (Fig. 1A) decreased from 3.3 × 3.0 × 3.1 (Fig. 1B) and pancreatic tail atrophy, without evidence of new metastatic lesions, indicating an adequate treatment response and disease stabilization with ICI therapy. This was presumed to be new-onset type 2 diabetes mellitus, and metformin 500 mg twice daily was started.

Two months later, he was readmitted with persistent nausea and vomiting. Laboratory studies marked the diagnosis of diabetic ketoacidosis (DKA), with hyperglycemia of 361 mg/dL (SI: 20 mmol/L), metabolic acidosis pH of 7.25, bicarbonate of 17 mEq/L (reference range: 22-29 mEq/L [SI: 22-29 mmol/L]), and elevated beta-hydroxybutyrate of 5.67 mmol/L (reference range: 0.02-0.27 mmol/L), requiring an insulin drip and later transition to a basal-bolus insulin regimen. Despite the rapid progression of diabetes, a repeat CT abdomen showed prior stable pancreatic findings. The C-peptide level was low at 0.1 ng/mL (SI: 0.033 nmol/L), raising concern for insulin-dependent diabetes mellitus. Endocrinology was consulted, metformin was discontinued, and the patient was eventually discharged on basal and prandial insulin.

**Figure 2:**
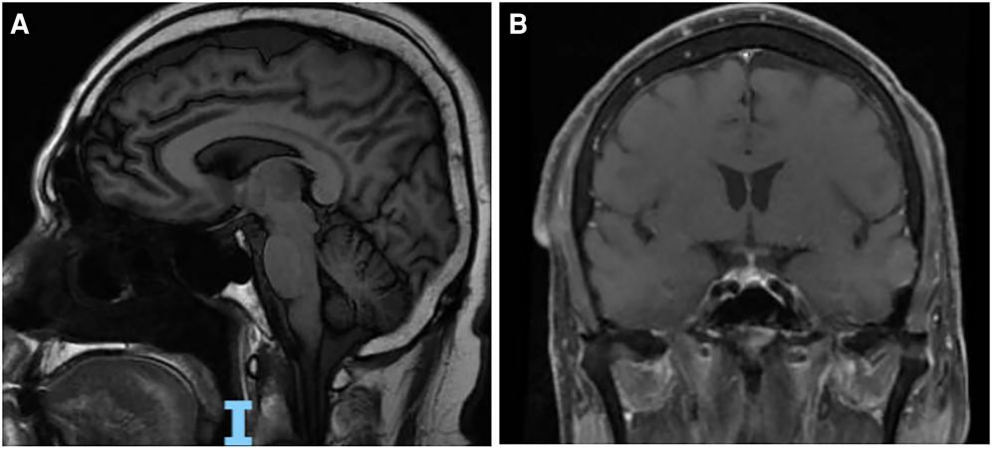
Brain MRI without any evidence of metastasis or hypophysitis.

Ten months into ICI therapy, he presented with confusion, lethargy, decreased oral intake, and orthostatic dizziness. Laboratory evaluation revealed hypoglycemia with a blood glucose of 54 mg/dL (SI: 3.0 mmol/L), low morning cortisol of 1.3 µg/dL (SI: 35.88 nmol/L) (reference range, 5.0-23.0 µg/dL [SI: 140-635 nmol/L]), and undetectable adrenocorticotropic (ACTH) of <5.0 pg/mL (SI: 1.1 pmol/L) (reference range, 0.0-46.0 pg/mL [SI: 2-11 pmol/L]), suggestive of secondary AI. No significant electrolyte derangements were noted, further supporting secondary AI. The ACTH stimulation test was not performed due to extremely low cortisol levels. A magnetic resonance imaging (MRI) scan of the brain revealed no evidence of pituitary metastasis or hypophysitis, as shown in Fig. 2.

## Treatment

The patient was started on hydrocortisone 20 mg in the morning and 10 mg in the evening for secondary AI.

## Outcome and follow-up

**Figure 3:**
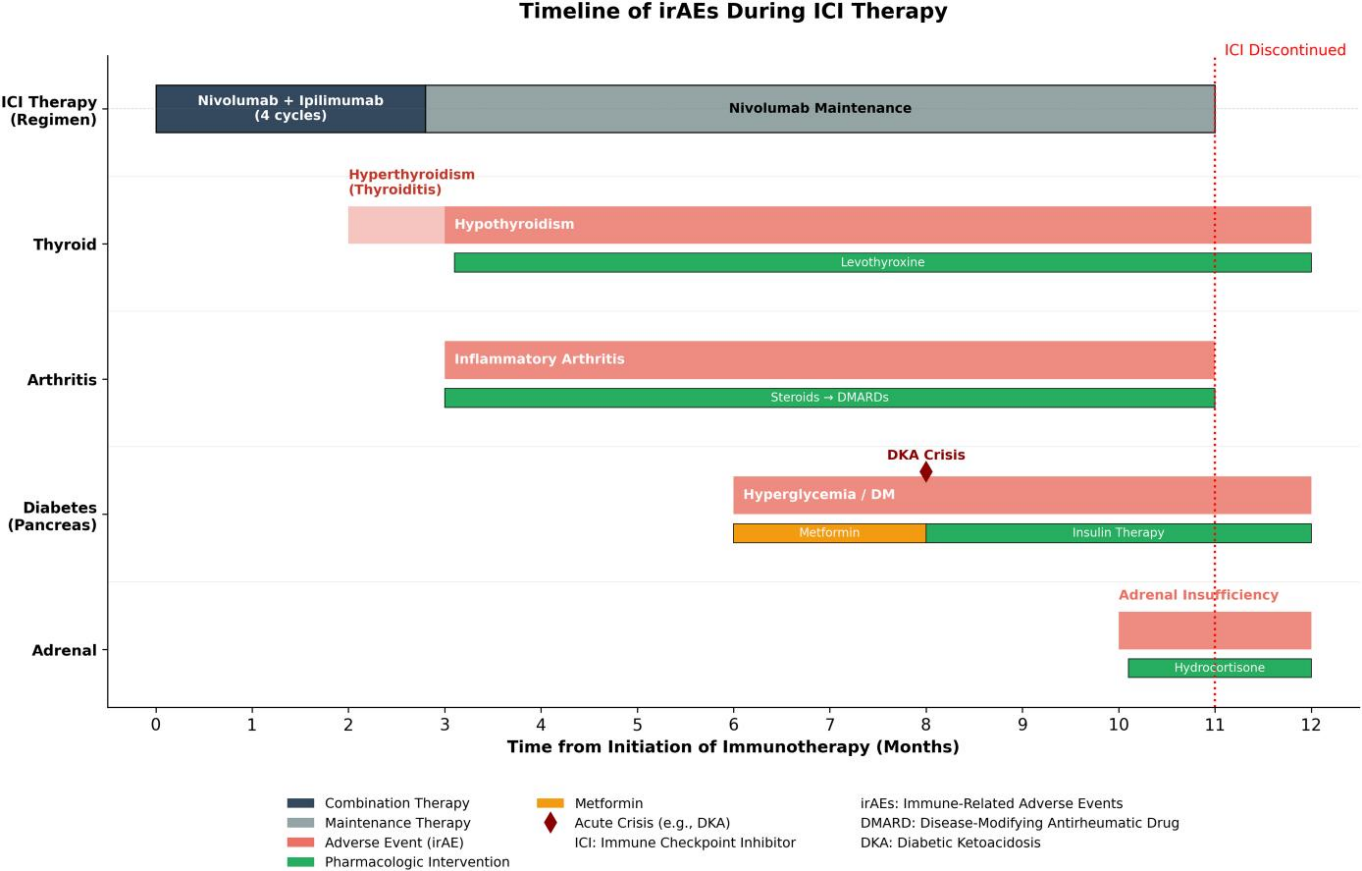
Graph showing the timeline of irAE in relation to immune check point inhibitor (ICI) therapy.

Eleven months into the ICI therapy, nivolumab was discontinued. The patient gradually improved, with decreased but persistent need for hydrocortisone and insulin. His immunosuppressive therapies (hydroxychloroquine and methotrexate) were discontinued following resolution of inflammatory symptoms. He underwent targeted radiation for the pancreatic metastasis and was maintained on levothyroxine, hydrocortisone, and insulin for the management of ICI-induced hypothyroidism, secondary AI, and diabetes mellitus. Table 1 summarizes irAE events, key laboratory findings, interventions, and outcomes after ICI therapy. Figure 3 represents a timeline of irAEs and their management in relation to ICI therapy.

**Table 1:** irAE and their outcomes

irAE event	Onset (month)	Key labs and findings	Reference range	Interventions	Outcomes
Thyroiditis →hypothyroidism	2-3	FT4: 6.3 ng/dL (SI: 81.1 pmol/L) → 0.2 ng/dL (SI: 2.57 pmol/L), TSH: 0.01→33.38 µIU/mL Anti-TPO antibody: 44 U/mL (SI: 44 U/mL)	FT4: 0.9-1.8 ng/dL (SI: 11.6-23.2 pmol/L) TSH: 0.55-4.78 µIU/mL (SI: 0.4-4.0 mIU/L) Anti-TPO antibody: 0-60 U/mL (SI: 0-60 U/mL)	Levothyroxine 100 mcg daily	Persistent hypothyroidism, continued levothyroxine replacement
Immune-mediated inflammatory arthritis	3	ESR 87 mm/hour (SI: 87 mm/hour), CRP 81.5 mg/L (SI: 81.5 mg/L); negative RF, ANA, anti-CCP, anti-dsDNA	ESR: 0-20 mm/hour (SI: 0-20 mm/hour) CRP: 0-8 mg/L (SI: 0-8 mg/L)	IV dexamethasone, followed by oral prednisone taper, then hydroxychloroquine and methotrexate	Symptom resolution; immunosuppressants stopped after ICI discontinuation
Insulin-dependent diabetes mellitus (with DKA)	6-8	Hyperglycemia 458 mg/dL (SI: 25.4 mmol/L) →361 mg/dL (SI: 20 mmol/L); HbA1c 8.4% (SI: 68 mmol/mol) C-peptide initially 2.4 ng/mL (SI: 0.79 nmol/L) →0.1 ng/mL (SI: 0.033 nmol/L); antiglutamic acid antibodies negative	Blood glucose: 60-140 mg/dL (SI: 4.0-5.6 mmol/L) Hba1c: 4.2-5.6% (SI: 20-42 mmol/mol) C-peptide: 0.8-3.8 ng/mL (SI: 0.26-1.3 nmol/L)	Metformin initially, then insulin drip and basal-bolus insulin; metformin discontinued	Persistent insulin dependence
Secondary adrenal insufficiency	10	Low morning cortisol 1.3 µg/dL (SI: 35.88 nmol/L); ACTH <5.0 pg/mL (SI: < 1.1 pmol/L) No hypophysitis on MRI	Cortisol: 5.0-23.0 µg/dL (SI: 140-635 nmol/L) ACTH: 0.0-46.0 pg/mL (SI: 2-11 pmol/L)	Hydrocortisone 20 mg in the AM + 10 mg in the PM	Clinical improvement; continued hydrocortisone replacement
ICI discontinuation	11	ND		Stopped nivolumab; stopped immunosuppressants after arthritis resolution	Gradual clinical improvement

Abbreviations: ACTH, adrenocorticotropic hormone; AM, morning; ANA, antinuclear antibody; Anti-CCP, anti-cyclic citrullinated peptide antibodies; Anti-dsDNA, anti-double-stranded deoxyribonucleic acid antibodies; Anti-TPO, antithyroid peroxidase antibodies; CRP, C-reactive protein; DKA, diabetic ketoacidosis; ESR, erythrocyte sedimentation rate; FT4, free thyroxine; HbA1c, hemoglobin A1c; ICI, immune-checkpoint inhibitor; irAE, immune-related adverse event; IV, intravenous; MRI, magnetic resonance imaging; ND, no data; PM, evening; RF, rheumatoid factor; TSH, thyroid-stimulating hormone.

## Discussion

ICI are established therapies for metastatic clear-cell RCC and other malignancies due to their ability to elicit robust antitumor immune responses [1, 2]. However, immune activation is associated with a tendency for immune-mediated destruction of healthy tissues, leading to a range of irAEs. Endocrine irAEs are among the most clinically significant due to their potential for morbidity and chronicity [3-5].

Several case reports have described ICI therapy causing 1 or 2 endocrinopathies, but the occurrence of 3 distinct endocrine irAEs in a single patient is exceedingly rare [6, 7]. Our literature review identified only 1 prior case in the United States reporting the triad of insulin-dependent diabetes, AI, and thyroiditis/hypothyroidism [8]. Thus, our case represents the second reported instance in the United States. A similar case has also been reported in Japan, highlighting the potential for multiglandular endocrine irAEs [9].

The incidence and timing of these endocrinopathies vary, typically occurring from weeks to months after ICI initiation, though delayed cases are reported. The most common endocrine irAEs include hypothyroidism, hypophysitis, insulin-dependent diabetes mellitus, and AI [5, 10].

Thyroid dysfunction is one of the most common endocrine irAEs, occurring in up to 16% of patients receiving combination ICI therapy. The evolution from transient hyperthyroidism to persistent hypothyroidism, as seen here, is typical of T-cell-mediated destructive thyroiditis rather than antibody-driven disease, consistent with the normal anti-TPO antibody levels, and often requires lifelong thyroid hormone replacement [5, 11].

The development of insulin-dependent diabetes and DKA ∼6 months into ICI therapy was notable for its abrupt and severe onset, marked by rapid beta-cell destruction with a sharp decline in C-peptide despite negative autoantibody testing. ICI-induced diabetes is a rare but increasingly recognized complication, occurring in ∼0.2-1.0% of patients, most associated with anti-PD-1 and anti-programmed death-ligand 1 (PD-L1) agents. Although it clinically resembles type 1 diabetes with sudden insulin deficiency from immune-mediated islet destruction, the absence of pancreatic autoantibodies, as seen in this patient, suggests a distinct immune dysregulation rather than classic autoimmunity [12]. Notably, his C-peptide levels remained undetectable 3 months after the DKA episode.

The subsequent diagnosis of secondary AI underscores the multisystem nature of ICI toxicities. Hypophysitis and primary AI are more frequently observed with anti-CTLA-4 agents (eg, ipilimumab) due to their preferential immunologic effects on the pituitary and adrenal glands, leading to immune-mediated inflammation and subsequent AI [13, 14]. Although hypophysitis is a well-recognized condition, particularly with CTLA-4 blockade, this patient had unremarkable pituitary MRI findings and no significant electrolyte abnormalities, suggesting AI. Low ACTH and cortisol levels, with clinical improvement on hydrocortisone, suggest isolated corticotroph dysfunction without radiographic hypophysitis, representing a possible atypical form of ICI-induced pituitary dysfunction that warrants close endocrine follow-up.

Managing irAEs and deciding whether to continue ICI therapy in patients with multiple endocrine irAEs is challenging. Adverse events are graded according to the Common Terminology Criteria for Adverse Events (CTCAE). Current guidelines recommend that controlled endocrinopathies do not require ICI discontinuation, as endocrine irAEs result from irreversible gland destruction that persists regardless of treatment cessation. Unlike grade 3-4 nonendocrine irAEs (pneumonitis, colitis, and hepatitis) that require immediate cessation due to life-threatening organ failure risk, the oncologic benefit of continued immunotherapy outweighs the risk of managed endocrinopathies with adequate hormone replacement [11, 15, 16]. This framework supported continuing ICI through hypothyroidism, inflammatory arthritis, and insulin-dependent diabetes with DKA, all managed with appropriate replacement or immunosuppressive therapy, with serial CT imaging confirming stable disease. ICI discontinuation became necessary only after an acute adrenal crisis occurred, which European Society of Endocrinology guidelines identify as a rare exception requiring endocrine stabilization before resuming therapy [17]. After 11 months of ICI therapy, nivolumab was discontinued following multidisciplinary consultation among oncology, endocrinology, and rheumatology specialists due to cumulative multisystem toxicity, lifelong multihormone replacement requirements, and the availability of alternative local therapy (stereotactic radiation) for a radiographically stable, oligometastatic lesion. This case illustrates the complex decision-making required to balance antitumor efficacy with management of potentially life-threatening immune toxicities.

## Learning points

This case emphasizes several important clinical lessons.

- Endocrine irAEs may present in a delayed and/or sequential manner, requiring vigilance and long-term monitoring during ICI therapy.
- The absence of autoantibodies does not exclude ICI-induced endocrinopathies, which often arise from direct T-cell-mediated tissue injury.
- Multidisciplinary collaboration among oncology, endocrinology, and rheumatology is essential to optimize outcomes, as management often requires immunosuppression, endocrine replacement, and modification of oncologic therapy.

## Contributors

All authors made individual contributions to the authorship. R.V. and O.O. were involved in the clinical assessment, diagnosis, and follow-up of the patient, as well as the preparation and submission of the manuscript. A.Y. contributed to the literature review and discussion. M.Q. was involved in data collection, diagnostic assessment, and literature review. All authors reviewed and approved the final draft.

## Data Availability

Data sharing is not applicable to this article as no datasets were generated or analyzed during the current study.
